# Ischemic Stroke as an Initial Manifestation of Silent, Evolved Myocardial Infarction: A Case Report

**DOI:** 10.7759/cureus.100631

**Published:** 2026-01-02

**Authors:** João Lagarteira, Rita Pera, Sara Sá, Antonio G Novais, Andrés Carrascal

**Affiliations:** 1 Internal Medicine Department, Unidade Local de Saúde do Nordeste, Bragança, PRT; 2 Internal Medicine and Intensive Care Department, Unidade Local de Saúde do Nordeste, Bragança, PRT

**Keywords:** antithrombotic therapy, cardioembolism, ischemic stroke, multidisciplinary approach, silent myocardial infarction

## Abstract

Acute ischemic stroke (AIS) is a rare initial manifestation of silent, evolved myocardial infarction (MI). AIS presents significant diagnostic and therapeutic challenges, particularly in patients with multiple comorbidities. Herein, we report a 64-year-old male patient with a history of hypertension and type 2 diabetes, who was admitted due to sudden neurological deficits without chest pain. The initial workup revealed an Alberta Stroke Program Early CT Score (ASPECTS) of 10/10 and no hypodense areas. Further investigations showed an ST-elevation MI without typical symptoms, indicating that a silent, evolved MI was the underlying cause. The 24-hour repeat head CT scan revealed a recent right lenticuloradial ischemic infarct. Multidisciplinary management focused on risk stratification, balancing antithrombotic therapy, and initiating early rehabilitation. This case emphasizes the importance of considering silent MI in AIS of unclear etiology and the need for individualized, multidisciplinary care.

## Introduction

Silent myocardial infarction (SMI), defined as objective evidence of myocardial necrosis in the absence of recognized symptoms, accounts for 20%-40% of all myocardial infarctions (MIs) [1,2]. SMI is frequently underdiagnosed in individuals with diabetes, autonomic dysfunction, or multiple cardiovascular risk factors. These patients do not often present with chest pain or other classic ischemic symptoms. Thus, SMI may progress unnoticed until the development of complications, including ventricular dysfunction, arrhythmias, and thrombus formation [1-3].

Cardioembolic stroke is a recognized complication of acute or chronic MI, particularly in the presence of left ventricular (LV) akinesia or aneurysm formation, which can promote intracavitary thrombus formation [4-6]. Cardioembolism accounts for up to 20%-30% of ischemic strokes, and MI-related thrombus is a known but infrequent cause [7]. If stroke occurs as the initial clinical manifestation of an unrecognized MI, diagnostic uncertainty may delay appropriate cardiac evaluation and treatment.

Simultaneous acute ischemic stroke (AIS) and acute myocardial infarction (AMI) or stroke secondary to evolving MI poses significant management challenges. The competing need for prompt reperfusion, anticoagulation, and antiplatelet therapy must be balanced against the risk of hemorrhagic transformation, particularly in the early phase after a large cerebral infarction [8-10]. Treatment guidelines offer limited guidance for these complex scenarios, emphasizing individualized, multidisciplinary decision-making.

Herein, we report a patient who presented with AIS as the initial manifestation of a silent, evolved ST-elevation MI complicated by LV thrombus. This case underscores the importance of considering SMI in the differential diagnosis of stroke of unclear etiology and shows the clinical reasoning required to manage the intricate balance of antithrombotic therapy, hemodynamic stability, and neurologic safety.

## Case presentation

A 64-year-old male patient who could independently perform activities of daily living presented to the emergency department due to sudden left upper-limb weakness (grade 4/5), mild left-sided mouth commissure deviation, and mild dysarthria. The patient denied chest pain, dyspnea, or other systemic symptoms. His previous medical history included hypertension and type 2 diabetes. He was managed with medications, including angiotensin receptor blockers, thiazide diuretics, calcium-channel blockers, insulin, and sodium-glucose cotransporter-2 inhibitors.

Upon admission, the patient’s vital signs were as follows: heart rate of 95 beats per minute, blood pressure of 128/68 mmHg, afebrile temperature, and oxygen saturation (SpO2) on room air of 94%.

Neurological examination revealed that the patient was alert and oriented, and the pupils were equal, symmetrical, and reactive to light. Mild dysarthria was noted, with no evidence of aphasia. No photophobia was noted, and meningeal signs were absent. No apparent sensory deficits were identified. Slight deviation of the oral commissure to the left was observed. Cranial nerve examination was unremarkable. No cerebellar deficits were detected. Upper limb strength was grade 4 on the left and grade 5 on the right. Lower limb strength was preserved bilaterally (grade 5). Gait was not assessed. The patient's National Institutes of Health Stroke Scale (NIHSS) score was 3 [11].

**Figure 1:**
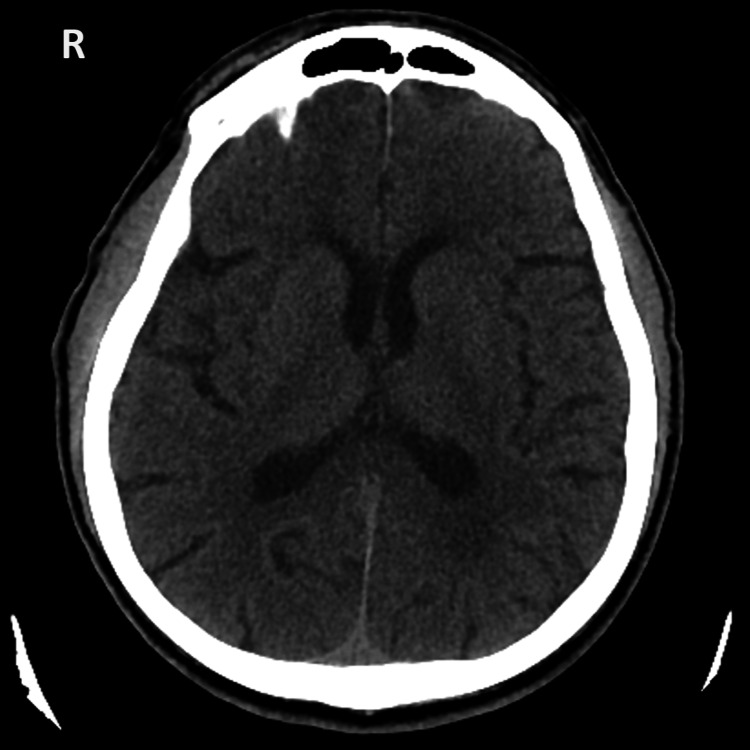
Admission head CT scan. Axial view showing no signs of lesions.

Upon admission, the head computed tomography (CT) scan (Figure 1) showed an Alberta Stroke Program Early CT Score (ASPECTS) of 10/10, and no hypodense areas or hemorrhage [12]. The CT angiography showed no large vessel occlusion or ischemic areas.

He was not eligible for thrombolysis or thrombectomy due to the low NIHSS score, mild and non-disabling symptoms, and no large vessel oclusion was identified.

**Figure 2:**
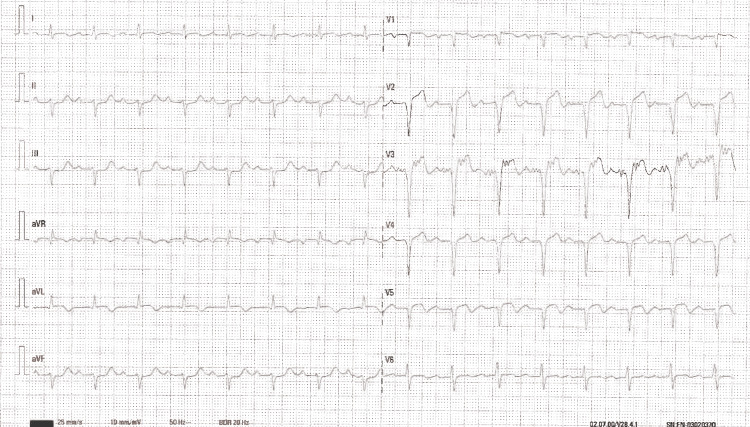
Electrocardiogram upon admission. Electrocardiogram revealed sinus rhythm, first-degree atrioventricular block, ST-segment elevation in V1-V5, augmented vector right (aVR), and augmented vector left (aVL), and ST-segment depression in II, III, and augmented vector foot (aVF).

Electrocardiogram revealed sinus rhythm, first-degree atrioventricular (AV) block, ST-segment elevation in V1-V5, augmented vector right (aVR), and augmented vector left (aVL), and ST-segment depression in II, III, and augmented vector foot (aVF) (Figure 2). These electrocardiographic findings were consistent with an evolving anterior myocardial infarction.

Laboratory findings are summarized in Table 1. High-sensitivity troponin I level was 1,724.4 ng/L (normal range: <34.6 ng/L), glucose level was 203 mg/dL (normal range: 74-106 mg/dL), and low-density lipoprotein level was 130 mg/dL (normal range: <155 mg/dL). The other results were unremarkable.

**Table 1:** Laboratory test results on admission. ALP, alkaline phosphatase; ALT, alanine transaminase; AST, aspartate aminotransferase; CK, creatine kinase; CK-MB, creatine kinase-myocardial band; CRP, C-reactive protein; GGT, gamma-glutamyl transferase; HBV, hepatitis B virus; HCV, hepatitis C virus; HDL, high density lipoprotein; HIV, human immunodeficiency virus; hsTnI, high sensitivity troponin I; INR, international normalized ratio; LDH, lactate dehydrogenase; LDL, low-density lipoprotein; TSH, thyroid-stimulating hormone; T4, thyroxine.

Parameters	Patient values	Reference range
Hemoglobin (g/dL)	14.7	12.3-15.3
Total leucocyte count (x10^9^/L)	5.02	4.4-11.3
Platelet count (x10^9^/L)	136	150-450
Sodium (mEq/L)	136	137-145
Potassium (mEq/L)	4.4	3.5-5.1
Chloride (mEq/L)	104	98-107
Glucose (mg/dL)	203	74-106
Urea (mg/dL)	39	17-43
Creatinine (mg/dL)	1.0	0.66-1.09
ALT (U/L)	43	<45
AST (U/L)	29	<35
Total bilirubin (mg/dL)	0.33	0.3-1.2
Direct bilirubin (mg/dL)	0.07	<0.2
ALP (U/L)	63	30-120
GGT (U/L)	30	<38
LDH (U/L)	236	<248
CK (U/L)	219	<145
CK-MB (U/L)	36	<=24
INR	0.97	-
CRP (mg/dL)	1.29	<0.1
hsTnI (ng/L)	1724.4	<34.6
Total cholesterol (mg/dL)	180	<200
Triglycerides (mg/dL)	95	<150
HDL (mg/dL)	31	30-60
LDL (mg/dL)	130	<155
Vitamin B12 (pg/mL)	414	187-883
Folic acid (ng/mL)	11.1	3.1-20.5
TSH (uUI/mL)	1.45	0.35-4.94
Free T4 (ng/dL)	0.88	0.7-1.48
HIV	Negative	-
HBV	Negative	-
HCV	Negative	-

**Figure 3:**
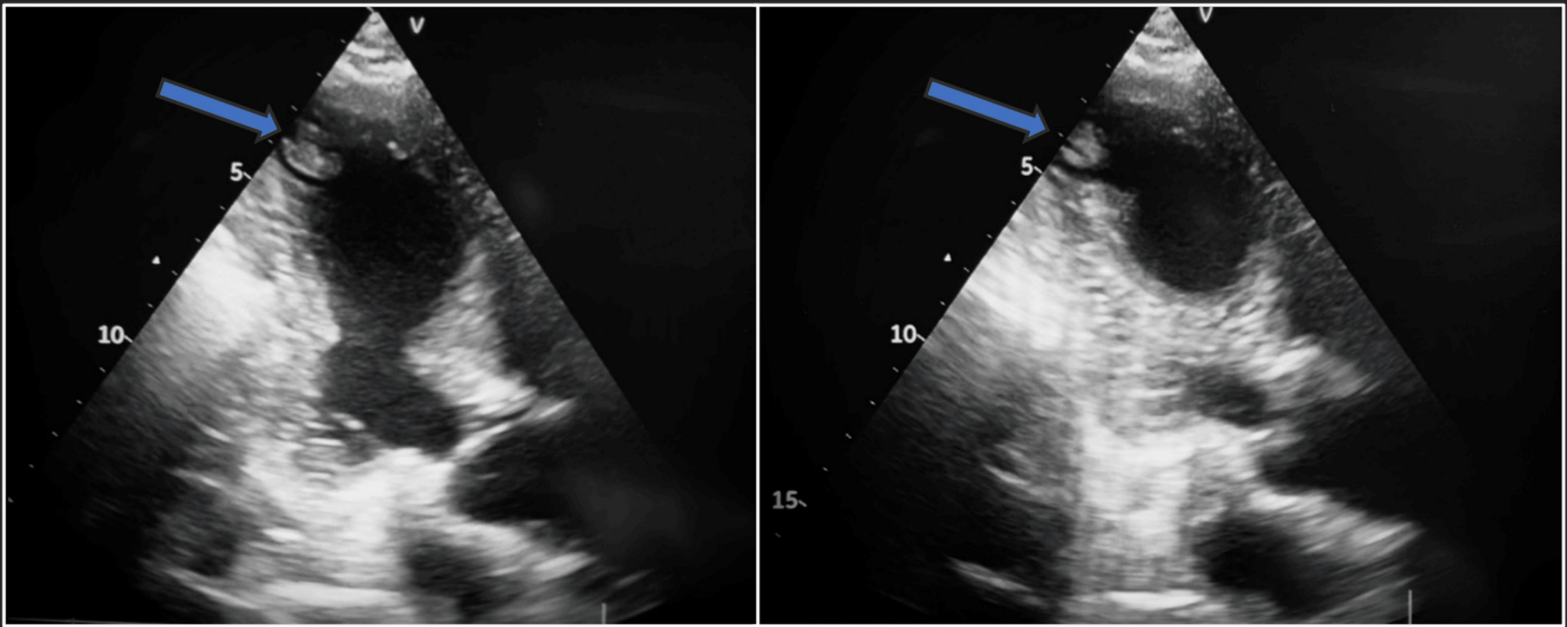
Bedside echocardiogram. Apical three-chamber view showing a thrombus on the apex of the left ventricle.

Bedside echocardiogram revealed akinesia of the interventricular septum and inferior wall, and apical thrombus in the left ventricle (Figure 3).

He was admitted to the stroke unit, and the patient's neurological deficits remained mild. After a multidisciplinary discussion (neurology/cardiology), the patient was started on dual antiplatelet therapy (aspirin + clopidogrel loading), high-intensity statin therapy, an angiotensin-converting enzyme inhibitor, glycemic control, and optimization of heart failure.

Dual antiplatelet therapy (DAPT) was chosen despite the cardioembolic nature of the ischemic stroke, most likely secondary to a left ventricular thrombus, because management of the evolved myocardial infarction required DAPT during the acute phase. In parallel, definitive treatment of the presumed stroke etiology necessitated systemic anticoagulation; however, given the recent ischemic event and the low NIHSS score, anticoagulation could only be safely initiated after the first 24 hours. Accordingly, DAPT was started on day one, following the joint neurology-cardiology consensus.

**Figure 4:**
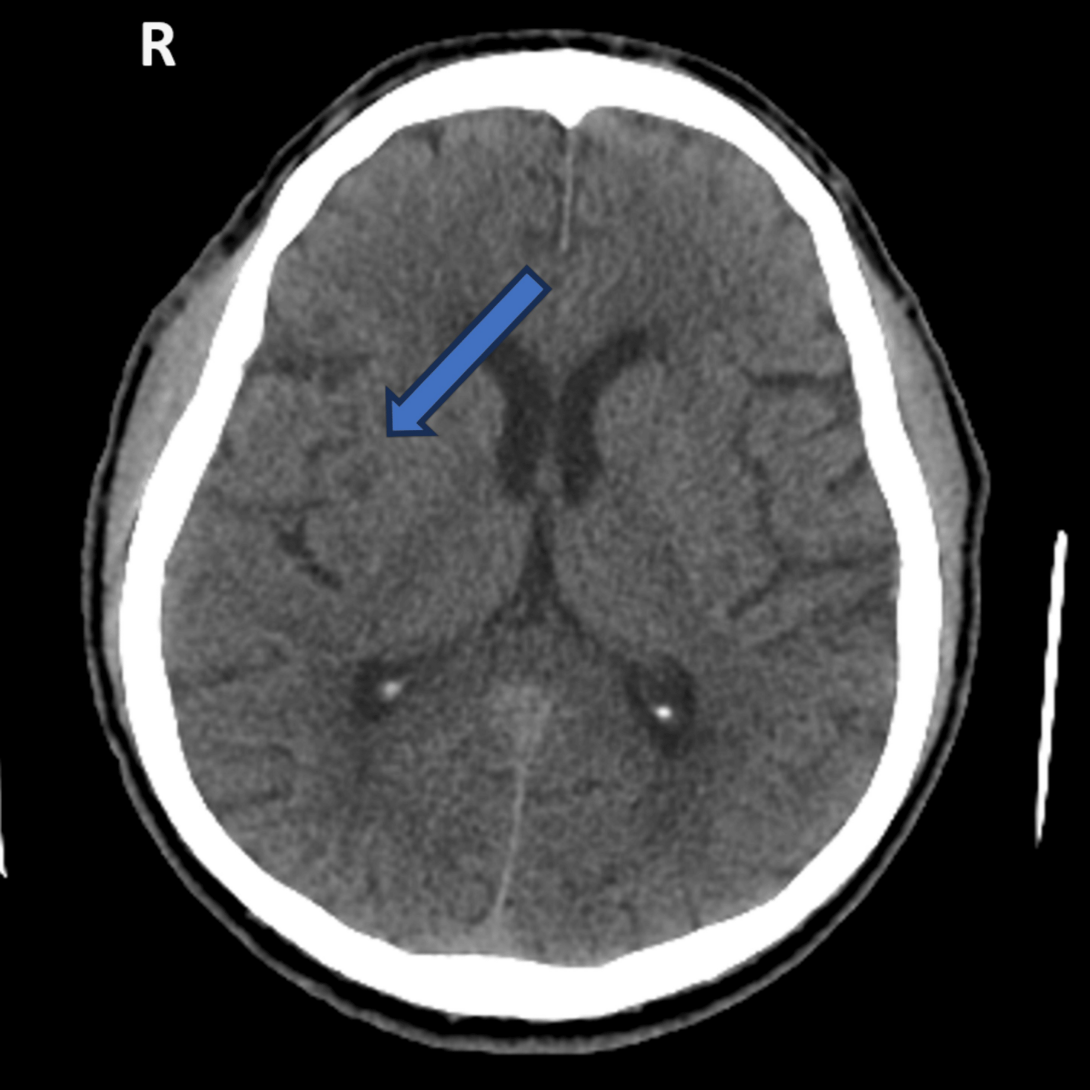
Twenty-four-hour repeat head CT scan. Axial view showing the right lenticuloradial hypodensity, consistent with recent ischemic stroke.

Repeat head CT imaging at 24 hours revealed a right lenticuloradial hypodensity, consistent with a recent ischemic lesion, and no hemorrhagic signs (Figure 4). Carotid Doppler ultrasonography revealed no significant lesions.

After confirmation of the absence of hemorrhagic complications and following joint neurology-cardiology consensus, therapy was de-escalated on day two to anticoagulation plus single antiplatelet therapy (SAPT).

The comprehensive echocardiogram was performed one week later, showing inferolateral hypokinesia, left ventricular ejection fraction of 41%, and no intracavitary thrombi.

The patient started physiotherapy with gradual neurological improvement, and he was discharged, with transition to a direct oral anticoagulant, for three to six months, with follow-up in cardiology and neurology.

## Discussion

This case emphasizes the diagnostic challenges in silent, evolved MI with AIS as the initial clinical manifestation. The patient denied chest pain. Thus, the underlying cardiac event would likely have been missed without systematic evaluation prompted by electrocardiogram abnormalities and elevated troponin levels. In particular, SMI is prevalent in individuals with diabetes and autonomic dysfunction, which is consistent with the patient’s profile [1-3].

Cardioembolic stroke secondary to LV thrombus remains a well-recognized complication of anterior MI, with thrombus formation occurring in up to 5%-15% of anterior ST-elevation MI cases despite modern reperfusion therapy [4-6]. Additional studies also highlight the role of LV thrombus as a cause of embolic stroke after MI [13,14]. The patient’s imaging findings, septal and inferior akinesia with an apical thrombus, strongly supported a cardioembolic mechanism. This etiology further reinforced the absence of atrial fibrillation during monitoring and a negative carotid evaluation.

Therapeutic management required a careful balance between competing ischemic and hemorrhagic risks. On one hand, the patient had an evolved MI with an LV thrombus conferring a high embolic risk; on the other, he had an acute cerebral infarction with an inherent risk of hemorrhagic transformation. The optimal timing of anticoagulation initiation in this context remains controversial. However, current guidelines suggest that in patients with a clearly identified cardioembolic source and high embolic risk, therapeutic anticoagulation may be considered after the first 24 hours, provided that serial neuroimaging excludes hemorrhagic transformation, particularly in patients with mild neurological deficits [8-10].

In this case, these principles guided a staged antithrombotic strategy. DAPT was initiated during the first 24 hours to address the acute coronary indication, as recommended in the management of myocardial infarction [8], while deferring anticoagulation due to the recent ischemic stroke. Following neurological reassessment and confirmation of a low NIHSS score without hemorrhagic transformation, therapy was de-escalated to systemic anticoagulation combined with SAPT. This approach allowed for simultaneous mitigation of coronary and embolic risks while minimizing hemorrhagic complications, consistent with strategies described in similar cases [13].

Multidisciplinary coordination was essential. Neurologists, cardiologists, and radiologists contributed to individualized decision-making regarding antithrombotic timing, monitoring, and rehabilitation. Early mobilization and secondary prevention are important for functional recovery and reduction of recurrent events [10,15].

## Conclusions

AIS can be an initial clinical manifestation of a silent, evolved MI. In patients with atypical or absent chest pain, particularly those with diabetes or multiple cardiovascular risk factors, clinicians should maintain a high index of suspicion for silent MI. A multidisciplinary evaluation and individualized balancing of ischemic and hemorrhagic risks are fundamental to optimizing outcomes. In this case, early cardiac evaluation, timely initiation of anticoagulation when appropriate, and structured rehabilitation contributed to a favorable recovery.
